# Recurrent macrophage activation syndrome since toddler age in a patient with HLA B27 positive juvenile ankylosing spondylitis

**DOI:** 10.1186/1546-0096-12-S1-P219

**Published:** 2014-09-17

**Authors:** Soo Young Lee, Jung Woo Rhim, Seung Beom Han, Jae Wook Lee, Nack-Gyun Chung, Jin Han Kang, Dae-Chul Jeong

**Affiliations:** 1 Pediatrics, College of Medicine, The Catholic University of Korea, Incheon, Korea, Republic of; 2 Pediatrics, College of Medicine, The Catholic University of Korea, Daejeon, Korea, Republic of; 3 Pediatrics, College of Medicine, The Catholic University of Korea, Seoul, Korea, Republic of

## Introduction

Recurrent macrophage activation syndrome (MAS) is very rare. Recurrent MAS should be considered secondary cause including autoimmune disease. We experienced recurrent MAS since toddler age without definite etiologies. We reported a case of recurrent MAS in 16 year-old boy with HLA B27 positive juvenile ankylosing spondylitis.

## Objectives

Recurrent MAS since toddler age is considered secondary autoimmune disease.

## Methods

A 16 year-old boy was transferred from the department of surgery due to remittent fever with pancytopenia and splenomegaly despite improvement of septic shock after intravenous immunoglobulin (IVIG) and antithrombin III. He had received fistulectomy with colostomy because of intractable perianal abscess 2 months previously. He had been diagnosed with hemophagocytic lymphohistiocytosis (HLH) according to HLH 1994 guideline at 3 years of age and had been treated with IVIG. HLH recurred 3 years later, and he was treated according to HLH 2004 protocol for 8 weeks, and remained symptom free without maintenance therapy. He relapsed again at ages 7 and 8, but we were unable to identify any causes. He received maintenance steroid treatment for 2 years after the 4^th^ attack. He remained symptom free until the development of back pain and right ankle joint pain with swelling at 15 years of age. He was diagnosed with juvenile ankylosing spondylitis compatible with bilateral active sacroilitis and positive HLA B27. He received naproxen with methotrexate, but showed symptom aggravation. He showed improvement after Etanercept, but suddenly developed intractable perianal abscess.

## Results

His laboratory data showed white blood cell count 1,240/μL, platelet 44,000/μL, ferritin 2,707ng/mL, triglyceride 343 mg/dL, aspatate aminotransferase 238 IU/L, alanine aminotransferase 145 IU/L, and fibrinogen 96 mg/dL. Bone marrow biopsy showed histiocytic hyperplasia with hemophagocytosis. There was no serologic evidence of any viral infections. He was treated with naproxen only, and improved without any other immunomodulatory medication. His laboratory data returned to near normal range within 4 weeks.

## Conclusion

In our case, underlying autoimmune disease should be considered as the cause of recurrent HLH in a young patient after familial HLH has been excluded.

## Disclosure of interest

None declared.

